# Supplementary material for: Hypotheses and evidence related to intense sweeteners and effects on appetite and body weight changes: A scoping review of reviews
Source: PLoS One. 2018 Jul 18;13(7):e0199558. doi: 10.1371/journal.pone.0199558 (PMC6051566; doi:10.1371/journal.pone.0199558)
Supplement: S1 Appendix — (PDF) [file pone.0199558.s001.pdf]

Ovid MEDLINE(R) Epub Ahead of Print, In-Process & Other Non-Indexed Citations, Ovid MEDLINE(R) Daily and Ovid MEDLINE(R) <1946 to Present>

#	**Searches**
1	Aspartame/
2	Cyclamates/
3	Saccharin/
4	Stevia/
5	Glycosides/
6	Sweetening Agents/
7	(acesulfam* or aspartam* or cyclam* or sacchar* or sucralose* or neohesperidin* or stevi* or glycosid* or neotame* or sweetening or sweetened or sweetener* or sugar substitute* or sugar free or non-nutritiv* or non-calori* or low-calori* or low-energy).tw.
8	or/1-7
9	Appetite Regulation/
10	Body Weight/
11	Body Weight Changes/
12	Overweight/
13	Weight Gain/
14	Body Mass Index/
15	Obesity/
16	Weight Loss/
17	((food* or eat*) adj3 (intake* or regulat* or control* or behavio*)).tw.
18	(appetite* or body weight* or overweight* or weight change* or weight reduc* or weight development* or weight gain* or weight loss* or body mass index or BMI or obesity or gut brain signal* or neuro-behavio* or neurobehavio*).tw.
19	or/9-18
20	8 and 19
21	limit 21 to "reviews (best balance of sensitivity and specificity)"

Embase <1974 to 2017 May 17>

#	**Searches**
1	acesulfame/
2	aspartame/
3	cyclamate sodium/
4	saccharin/
5	sucralose/
6	neohesperidin/
7	steviol/
8	glycoside/
9	Stevia/
10	neotame/
11	sweetening agent/

12	(acesulfam* or aspartam* or cyclam* or sacchar* or sucralose* or neohesperidin* or stevi* or glycosid* or neotame* or sweetening or sweetened or sweetener* or sugar substitute* or sugar free or non-nutritiv* or non-calori* or low-calori* or low-energy).tw.
13	or/1-12
14	appetite/
15	food intake/
16	body weight/
17	weight change/
18	obesity/
19	weight gain/
20	body mass/
21	weight reduction/
22	((food* or eat*) adj3 (intake* or regulat* or control* or behavio*)).tw.
23	(appetite* or body weight* or overweight* or weight change* or weight reduc* or weight development* or weight gain* or weight loss* or body mass index or BMI or obesity or gut brain signal* or neuro-behavio* or neurobehavio*).tw.
24	or/14-23
25	13 and 24
26	limit 25 to embase
27	limit 26 to "reviews (best balance of sensitivity and specificity)"

Cochrane Database of Systematic Reviews: to Issue 5 of 12, May 2017; Health Technology Assessment Database: to Issue 4 of 4, October 2016

ID	Search
#1	[mh ^aspartame]:ti,ab,kw Publication Year from 2006 to 2017, in Cochrane Reviews (Reviews and Protocols) (Word variations have been searched)
#2	[mh ^aspartame] Publication Year from 2006 to 2017, in Other Reviews and Technology Assessments (Word variations have been searched)
#3	[mh ^cyclamates]:ti,ab,kw Publication Year from 2006 to 2017, in Cochrane Reviews (Reviews and Protocols) (Word variations have been searched)
#4	[mh ^cyclamates] Publication Year from 2006 to 2017, in Other Reviews and Technology Assessments (Word variations have been searched)
#5	[mh ^saccharin]:ti,ab,kw Publication Year from 2006 to 2017 (Word variations have been searched)
#6	[mh ^saccharin] Publication Year from 2006 to 2017, in Other Reviews and Technology Assessments (Word variations have been searched)
#7	[mh ^stevia]:ti,ab,kw Publication Year from 2006 to 2017, in Cochrane Reviews (Reviews and Protocols) (Word variations have been searched)
#8	[mh ^stevia] Publication Year from 2006 to 2017, in Other Reviews and Technology Assessments (Word variations have been searched)
#9	[mh ^glycosides]:ti,ab,kw Publication Year from 2006 to 2017, in Cochrane Reviews (Reviews and Protocols) (Word variations have been searched)
#10	[mh ^glycosides] Publication Year from 2006 to 2017, in Other Reviews and Technology Assessments (Word variations have been searched)
#11	[mh ^"sweetening agents"]:ti,ab,kw Publication Year from 2006 to 2017, in Cochrane Reviews (Reviews and Protocols) (Word variations have been searched)
#12	[mh ^"sweetening agents"] Publication Year from 2006 to 2017, in Other Reviews and Technology Assessments (Word variations have been searched)
#13	(acesulfam* or aspartam* or cyclam* or sacchar* or sucralose* or neohesperidin* or stevi* or glycosid* or neotame* or sweetening or sweetened or sweetener* or "sugar substitute*" or "sugar free" or "non-

	nutritiv*" or "non-calori*" or "low-calori*" or "low-energy"):ti,ab,kw Publication Year from 2006 to 2017, in Cochrane Reviews (Reviews and Protocols) (Word variations have been searched)
#14	(acesulfam* or aspartam* or cyclam* or sacchar* or sucralose* or neohesperidin* or stevi* or glycosid* or neotame* or sweetening or sweetened or sweetener* or "sugar substitute*" or "sugar free" or "non-nutritiv*" or "non-calori*" or "low-calori*" or "low-energy") Publication Year from 2006 to 2017, in Other Reviews and Technology Assessments (Word variations have been searched)
#15	{or #1-#14}
#16	[mh ^"appetite regulation"]:ti,ab,kw Publication Year from 2006 to 2017, in Cochrane Reviews (Reviews and Protocols) (Word variations have been searched)
#17	[mh ^"appetite regulation"] Publication Year from 2006 to 2017, in Other Reviews and Technology Assessments (Word variations have been searched)
#18	[mh ^"body weight"]:ti,ab,kw Publication Year from 2006 to 2017, in Cochrane Reviews (Reviews and Protocols) (Word variations have been searched)
#19	[mh ^"body weight"] Publication Year from 2006 to 2017, in Other Reviews and Technology Assessments (Word variations have been searched)
#20	[mh ^"body weight changes"]:ti,ab,kw Publication Year from 2006 to 2017, in Cochrane Reviews (Reviews and Protocols) (Word variations have been searched)
#21	[mh ^"body weight changes"] Publication Year from 2006 to 2017, in Other Reviews and Technology Assessments (Word variations have been searched)
#22	[mh ^overweight]:ti,ab,kw Publication Year from 2006 to 2017 (Word variations have been searched)
#23	[mh ^overweight] Publication Year from 2006 to 2017, in Other Reviews and Technology Assessments (Word variations have been searched)
#24	[mh ^"weight gain"]:ti,ab,kw Publication Year from 2006 to 2017, in Cochrane Reviews (Reviews and Protocols) (Word variations have been searched)
#25	[mh ^"weight gain"] Publication Year from 2006 to 2017, in Other Reviews and Technology Assessments (Word variations have been searched)
#26	[mh ^"body mass index"]:ti,ab,kw Publication Year from 2006 to 2017, in Cochrane Reviews (Reviews and Protocols) (Word variations have been searched)
#27	[mh ^"body mass index"] Publication Year from 2006 to 2017, in Other Reviews and Technology Assessments (Word variations have been searched)
#28	[mh ^obesity]:ti,ab,kw Publication Year from 2006 to 2017, in Cochrane Reviews (Reviews and Protocols) (Word variations have been searched)
#29	[mh ^obesity] Publication Year from 2006 to 2017, in Other Reviews and Technology Assessments (Word variations have been searched)
#30	[mh ^"weight loss"]:ti,ab,kw Publication Year from 2006 to 2017, in Cochrane Reviews (Reviews and Protocols) (Word variations have been searched)
#31	[mh ^"weight loss"] Publication Year from 2006 to 2017, in Other Reviews and Technology Assessments (Word variations have been searched)
#32	((food* or eat*) near/3 (intake* or regulat* or control* or behavio*)):ti,ab,kw Publication Year from 2006 to 2017, in Cochrane Reviews (Reviews and Protocols) (Word variations have been searched)
#33	((food* or eat*) near/3 (intake* or regulat* or control* or behavio*)) Publication Year from 2006 to 2017, in Other Reviews and Technology Assessments (Word variations have been searched)
#34	(appetite* or "body weight*" or overweight* or "weight change*" or "weight reduc*" or "weight development*" or "weight gain*" or "weight loss*" or "body mass index" or BMI or obesity or "gut brain signal*" or "neuro-behavio*" or neurobehavio*):ti,ab,kw Publication Year from 2006 to 2017, in Cochrane Reviews (Reviews and Protocols) (Word variations have been searched)
#35	(appetite* or "body weight*" or overweight* or "weight change*" or "weight reduc*" or "weight development*" or "weight gain*" or "weight loss*" or "body mass index" or BMI or obesity or "gut brain signal*" or "neuro-behavio*" or neurobehavio*) Publication Year from 2006 to 2017, in Other Reviews and Technology Assessments (Word variations have been searched)
#36	{or #16-#35}
#37	#15 and #36

Epistemonikos:

(title:((acesulfam* OR aspartam* OR cyclam* OR sacchar* OR sucralose* OR neohesperidin* OR stevi* OR glycosid* OR neotame* OR sweetening OR sweetened OR sweetener OR "sugar substitute*" OR "sugar free" OR "non-nutritiv*" OR "non-calori*" OR "low-calori*" OR "low-energy") AND (appetite* OR "food intake*" OR eating OR "body weight*" OR overweight* OR "weight change*" OR "weight reduc*" OR "weight development*" OR "weight gain*" OR "weight loss*" OR "body mass index" OR BMI OR obesity OR "gut brain signal*" OR "neuro-behavio*" OR neurobehavio*)) OR abstract:((acesulfam* OR aspartam* OR cyclam* OR sacchar* OR sucralose* OR neohesperidin* OR stevi* OR glycosid* OR neotame* OR sweetening OR sweetened OR sweetener OR "sugar substitute*" OR "sugar free" OR "non-nutritiv*" OR "non-calori*" OR "low-calori*" OR "low-energy") AND (appetite* OR "food intake*" OR eating OR "body weight*" OR overweight* OR "weight change*" OR "weight reduc*" OR "weight development*" OR "weight gain*" OR "weight loss*" OR "body mass index" OR BMI OR obesity OR "gut brain signal*" OR "neuro-behavio*" OR neurobehavio*)))

Web of Science (core collection):

Set	Results	
# 5	**138**	#4 AND #1<br>*Indexes=SCI-EXPANDED, SSCI, A&HCI, ESCI Timespan=2006-2017*
# 4	**5,969**	#3 OR #2<br>*Indexes=SCI-EXPANDED, SSCI, A&HCI, ESCI Timespan=2006-2017*
# 3	**5,532**	(TS=(appetite* or "body weight*" or overweight* or "weight change*" or "weight reduc*" or "weight development*" or "weight gain*" or "weight loss*" or "body mass index" or BMI or obesity or "gut brain signal*" or "neuro-behavio*" or neurobehavio*)) *AND* **DOCUMENT TYPES:** (Review)<br>*Indexes=SCI-EXPANDED, SSCI, A&HCI, ESCI Timespan=2006-2017*
# 2	**877**	(TS=((food* or eat*) NEAR/3 (intake* or regulat* or control* or behavio*))) *AND* **DOCUMENT TYPES:** (Review)<br>*Indexes=SCI-EXPANDED, SSCI, A&HCI, ESCI Timespan=2006-2017*
# 1	**1,915**	(TS=(acesulfam* or aspartam* or cyclam* or sacchar* or sucralose* or neohesperidin* or stevi* or glycosid* or neotame* or sweetening or sweetemed or sweetener or "sugar substitute*" or "sugar free" or "non-nutritiv*" or "non-calori*" or "low-calori*" or "low-energy")) *AND* **DOCUMENT TYPES:** (Review)<br>*Indexes=SCI-EXPANDED, SSCI, A&HCI, ESCI Timespan=2006-2017*
