# Supplementary material for: Hypotheses and evidence related to intense sweeteners and effects on appetite and body weight changes: A scoping review of reviews
Source: PLoS One. 2018 Jul 18;13(7):e0199558. doi: 10.1371/journal.pone.0199558 (PMC6051566; doi:10.1371/journal.pone.0199558)
Supplement: S1 Table — (PDF) [file pone.0199558.s002.pdf]

Reference	Reason for exclusion
AlDeeb, O.A., H. Mahgoub, and N.H. Foda, Sucralose. Profiles of Drug Substances Excipients & Related Methodology, 2013. 38: p. 423-62.	Aim outside our scope
Aune, D., Soft drinks, aspartame, and the risk of cancer and cardiovascular disease. American Journal of Clinical Nutrition, 2012. 96(6): p. 1249-1251.	Not considered a review (editorial)
Bachman, C.M., T. Baranowski, and T.A. Nicklas, Is there an association between sweetened beverages and adiposity? Nutrition Reviews, 2006. 64(4): p. 153-174.	Aim outside our scope
Bell, D.S., Changes seen in gut bacteria content and distribution with obesity: causation or association? Postgraduate Medicine, 2015. 127(8): p. 863-8.	Aim outside our scope
Bellisle, F., et al., Sweetness, satiation, and satiety. Journal of Nutrition, 2012. 142(6): p. 1149S-54S.	Aim outside our scope
Benoit, S.C., J.F. Davis, and T.L. Davidson, Learned and cognitive controls of food intake. Brain Research, 2010. 1350: p. 71-76.	Aim outside our scope
Brahmini, M., et al., Myths and facts about aspartame and sucralose: A critical review. International Journal of Research in Ayurveda and Pharmacy, 2012. 3(3): p. 373-375.	Outcomes outside our scope
Bryant, C. and J. McLaughlin, Low calorie sweeteners: Evidence remains lacking for effects on human gut function. Physiology & Behavior, 2016. 164(Pt B): p. 482-5.	Aim outside our scope
Bukhamseen, F. and L. Novotny, Artificial sweeteners and sugar substitutes -some properties and potential health benefits and risks. Research Journal of Pharmaceutical, Biological and Chemical Sciences, 2014. 5(1): p. 638-649.	Aim outside our scope
Burls, A., et al., Drinking extra water or other non-caloric beverages for promoting weight loss or preventing weight gain. Cochrane Database of Systematic Reviews, 2016. 2016 (5) (no pagination)(CD012211).	Protocol for systematic review
Corkey, B.E., Diabetes: have we got it all wrong? Insulin hypersecretion and food additives: cause of obesity and diabetes? Diabetes Care, 2012. 35(12): p. 2432-7.	Aim outside our scope
Daniels, M.C. and B.M. Popkin, Impact of water intake on energy intake and weight status: a systematic review. Nutrition Reviews, 2010. 68(9): p. 505-21.	Aim outside our scope
Gardner, C., et al., Nonnutritive sweeteners: Current use and health perspectives: A scientific statement from the American heart association and the American diabetes association. Circulation, 2012. 126(4): p. 509-519.	The same article is co-publised in Diabetes care (included).
Greenwood, D.C., et al., Association between sugar-sweetened and artificially sweetened soft drinks and type 2 diabetes: systematic review and dose-response meta-analysis of prospective studies. British Journal of Nutrition, 2014. 112(5): p. 725-734.	Outcomes outside our scope
Grotz, V.L. and I.C. Munro, An overview of the safety of sucralose. Regulatory Toxicology & Pharmacology, 2009. 55(1): p. 1-5.	Outcomes outside our scope

Gupta, S., et al., Artificial sweeteners. JK Science, 2011. 14(1): p. 2-4.	Aim outside our scope
Hill, J.O., What do you say when your patients ask whether low-calorie sweeteners help with weight management? American Journal of Clinical Nutrition, 2014. 100(3): p. 739-740.	Not considered a review (editorial)
Imamura, F., et al., Consumption of sugar sweetened beverages, artificially sweetened beverages, and fruit juice and incidence of type 2 diabetes: Systematic review, meta-analysis, and estimation of population attributable fraction. BMJ (Online), 2015. 351 (no pagination)(h3576).	Outcomes outside our scope
Kellett, G.L., et al., Sugar absorption in the intestine: the role of GLUT2. Annual Review of Nutrition, 2008. 28: p. 35-54.	Aim outside our scope
Laffitte, A., F. Neiers, and L. Briand, Functional roles of the sweet taste receptor in oral and extraoral tissues. Current Opinion in Clinical Nutrition & Metabolic Care, 2014. 17(4): p. 379-85.	Aim outside our scope
Laviada-Molina, H., et al. Non-nutritive sweeteners for the prevention or treatment of being overweight or obesity. Cochrane Database of Systematic Reviews, 2016. DOI: 10.1002/14651858.CD012298.	Protocol for systematic review
Le, K.A., F. Robin, and O. Roger, Sugar replacers: from technological challenges to consequences on health. Current Opinion in Clinical Nutrition and Metabolic Care, 2016. 19(4): p. 310-315.	Outcomes outside our scope
Logue, C., et al., The potential application of a biomarker approach for the investigation of low-calorie sweetener exposure. Proceedings of the Nutrition Society, 2016. 75(2): p. 216-25.	Outcomes outside our scope
Low, Y.Q., K. Lacy, and R. Keast, The role of sweet taste in satiation and satiety. Nutrients, 2014. 6(9): p. 3431-50.	Aim outside our scope
Magnuson, B., Aspartame-facts and fiction. New Zealand Medical Journal, 2010. 123(1311): p. 53-57.	Not considered a review (commentary)
Magnuson, B.A., Burdock, G.A., Doull, J., et al. Aspartame: a safety evaluation based on current use levels, regulations, and toxicological and epidemiological studies. Crit Rev Toxicol. 2007;37(8):629-727.	Aim outside our scope
Magnuson, B.A., et al., Biological fate of low-calorie sweeteners. Nutrition Reviews, 2016. 74(11): p. 670-689.	Outcomes outside our scope
Mandrioli, D., C.E. Kearns, and L.A. Bero, Relationship between research outcomes and risk of bias, study sponsorship, and author financial conflicts of interest in reviews of the effects of artificially sweetened beverages on weight outcomes: A systematic review of reviews. PLoS ONE, 2016. 11 (9) (no pagination)(e0162198).	Outcomes outside our scope
Manuprakash, S.K. and K. Varadarajshenoy, Synthetic drinks and ill health in children. Indian Journal of Public Health Research and Development, 2012. 3(2): p. 116-119.	Aim outside our scope

Meyer-Gerspach, A.C., B. Wolnerhanssen, and C. Beglinger, Functional roles of low calorie sweeteners on gut function. Physiology & Behavior, 2016. 164(Pt B): p. 479-81.	Aim outside our scope
Mohd-Radzman, N.H., et al., Potential Roles of Stevia rebaudiana Bertoni in Abrogating Insulin Resistance and Diabetes: A Review. Evidence-Based Complementary & Alternative Medicine: eCAM, 2013. 2013: p. 718049.	Outcomes outside our scope
Mooradian, A.D., M. Smith, and M. Tokuda, The role of artificial and natural sweeteners in reducing the consumption of table sugar: A narrative review. Clinical Nutrition ESPEN, 2017. 18: p. 1-8.	Aim outside our scope
Murray, S., et al., Recent studies of the effects of sugars on brain systems involved in energy balance and reward: Relevance to low calorie sweeteners. Physiology & Behavior, 2016. 164(Pt B): p. 504-8.	Aim outside our scope
Myles, I.A., Fast food fever: reviewing the impacts of the Western diet on immunity. Nutrition Journal, 2014. 13: p. 61.	Aim outside our scope
Narain, A., C.S. Kwok, and M.A. Mamas, Soft drink intake and the risk of metabolic syndrome: Asystematic review and meta-analysis. International Journal of Clinical Practice, 2017. 71(2): p. 12.	Outcomes outside our scope
Narain, A., C.S. Kwok, and M.A. Mamas, Soft drinks and sweetened beverages and the risk of cardiovascular disease and mortality: a systematic review and meta-analysis. International Journal of Clinical Practice, 2016. 70(10): p. 791-805.	Outcomes outside our scope
Nseir, W., F. Nassar, and N. Assy, Soft drinks consumption and nonalcoholic fatty liver disease. World Journal of Gastroenterology, 2010. 16(21): p. 2579-2588.	Aim outside our scope
Pan, A., et al., Changes in water and beverage intake and long-term weight changes: results from three prospective cohort studies. International Journal of Obesity, 2013. 37(10): p. 1378-85.	Not considered a review (primary study)
Payne, A.N., C. Chassard, and C. Lacroix, Gut microbial adaptation to dietary consumption of fructose, artificial sweeteners and sugar alcohols: implications for host-microbe interactions contributing to obesity. Obesity Reviews, 2012. 13(9): p. 799-809.	Aim outside our scope
Qurrat ul, A. and S.A. Khan, Artificial sweeteners: safe or unsafe? JPMA - Journal of the Pakistan Medical Association, 2015. 65(2): p. 225-7.	Aim outside our scope
Renwick, A.G. and H. Nordmann, First European conference on aspartame: Putting safety and benefits into perspective. Synopsis of presentations and conclusions. Food and Chemical Toxicology, 2007. 45(7): p. 1308-1313.	Not considered a review (synopsis of conference presentations)
Renwick, A.G. and S.V. Molinary, Sweet-taste receptors, low-energy sweeteners, glucose absorption and insulin release. British Journal of Nutrition, 2010. 104(10): p. 1415-1420.	Aim outside our scope

Rippe, J.M. and L. Tappy, Sweeteners and health: Findings from recent research and their impact on obesity and related metabolic conditions. International Journal of Obesity, 2016. 40: p. S1-S5.	Aim outside our scope
Rizkalla, S.W., Health implications of fructose consumption: A review of recent data. Nutrition and Metabolism, 2010. 7(82).	Aim outside our scope
Rogers, P., et al., Systematic review: Low energy sweetener consumption, energy intake and body weight in animals and humans. Annals of Nutrition and Metabolism, 2015. 67: p. 339-339.	Not considered a review (conference abstract, later publication included)
Rogers, P.J., Effects of low-energy sweeteners consumption on appetite and weight control. Annals of Nutrition and Metabolism, 2015. 67: p. 96-97.	Not considered a review (conference abstract, later publication included)
Ruanpeng, D., et al., Sugar and artificially sweetened soda linked to obesity: A systematic review and meta-analysis. Endocrine Reviews, 2016.	Not considered a review (conference abstract, later publication included)
Salunkhe, V.R. and S.B. Bhise, Stevia rebaudiana: An alternative to synthetic sweeteners. Indian Drugs, 2010. 47(2): p. 5-13.	Outcomes outside our scope
Sharma, A., et al., Artificial sweeteners as a sugar substitute: Are they really safe? Indian Journal of Pharmacology, 2016. 48(3): p. 237-240.	Aim outside our scope
Sievenpiper, J.L., Low calorie sweeteners in weight loss: Friend or foe? Annals of Nutrition and Metabolism, 2015. 67: p. 99-100.	Not considered a review (conference abstract)
Simmons, A.L., J.J. Schlezinger, and B.E. Corkey, What Are We Putting in Our Food That Is Making Us Fat? Food Additives, Contaminants, and Other Putative Contributors to Obesity. Current Obesity Reports, 2014. 3(2): p. 273-285.	Aim outside our scope
Singh, G.M., Sugar sweetened beverages are associated with greater incidence of diabetes but there is a paucity of evidence on healthfulness of artificially-sweetened beverages and fruit juices. Evidence-Based Medicine, 2016. 21(1): p. 35.	Not considered a review (commentary)
Smeets, P.A., A. Erkner, and C. de Graaf, Cephalic phase responses and appetite. Nutrition Reviews, 2010. 68(11): p. 643-55.	Aim outside our scope
Stanhope, K.L., Sugar consumption, metabolic disease and obesity: The state of the controversy. Critical Reviews in Clinical Laboratory Sciences, 2016. 53(1): p. 52-67.	Aim outside our scope
Suez, J., et al., Non-caloric artificial sweeteners and the microbiome: findings and challenges. Gut Microbes, 2015. 6(2): p. 149-55.	Aim outside our scope
Swithers, S.E., A.A. Martin, and T.L. Davidson, High-intensity sweeteners and energy balance. Physiology & Behavior, 2010. 100(1): p. 55-62.	Aim outside our scope
Sylvetsky, A.C., J.E. Blau, and K.I. Rother, Understanding the metabolic and health effects of low-calorie sweeteners: methodological considerations and	Outcomes outside our scope

implications for future research. Reviews in Endocrine & Metabolic Disorders, 2016. 17(2): p. 187-94.	
Treesukosol, Y., K.R. Smith, and A.C. Spector, The functional role of the T1R family of receptors in sweet taste and feeding. Physiology & Behavior, 2011. 105(1): p. 14-26.	Aim outside our scope
Wang, D.D., et al., Creating a literature database of low-calorie sweeteners and health studies: evidence mapping. BMC Med Res Methodol, 2016. 16: p. 1.	Aim outside our scope
Wang, D.D., et al., Low-calorie sweeteners and health. FASEB Journal. Conference: Experimental Biology, 2015. 29(1).	Not considered a review (conference abstract)
Whitehouse, C.R., J. Boullata, and L.A. McCauley, The potential toxicity of artificial sweeteners. AAOHN Journal, 2008. 56(6): p. 251-9; quiz 260-1.	Aim outside our scope
Wijarnpreecha, K., et al., Associations of sugar-and artificially sweetened soda with nonalcoholic fatty liver disease: A systematic review and meta-analysis. Qjm, 2016. 109(7): p. 461-466.	Outcomes outside our scope
